# The Alloys of Gold for Dental Purposes

**Published:** 1852-01

**Authors:** James Robinson

**Affiliations:** Surgeon Dentist to the Royal Free Hospital, &c.


### ARTICLE VIII.

*The Alloys of Gold for Dental Purposes.* By JAMES ROBINSON, Surgeon Dentist to the Royal Free Hospital, &c.

THE public is much indebted to the *Lancet* for the series of papers published under the title of the Analytical Sanitary Commission, which prove by the aid of the microscope and chemical analysis that the common necessaries of life are largely adulterated—in some cases with cheaper articles of an innoxious character, but in others with substances absolutely of a poisonous nature. The latter are undoubtedly among the predisposing and exciting causes of disease, and often induce symptoms of a peculiar and anomalous character. The recognition of adulterations, therefore, falls within the immediate province of the medical press, considered as the guardian of the public health.

Among the adulterations we must rank the employment of alloys containing the baser metals in large proportions for dental purposes, the placing these alloys where they are exposed to the action of the secretions of the mouth and stomach, and atmospheric air, conditions most favorable for the oxydation and corrosion of the metals and the formation of salts, which being conveyed to the stomach with the saliva, exert an injurious influence on the health of the individual. In order to elucidate the corrosive action which takes place upon the alloys used as plates for artificial teeth, it is neeessary to say a few words on the natural and diseased conditions of the saliva and fluids of the stomach by which this action is excited.

The food in the act of chewing is mixed with the saliva, which being a viscid fluid, entangles a quantity of bubbles of atmospheric air, which are carried with it into the stomach. The saliva in its natural condition as it escapes from the ducts of the salivary glands is, a viscid, colorless, tasteless, and inodorous fluid, not quite transparent, and generally alkaline; but occasionally neutral. Healthy saliva like other animal fluids, varies in its amount of solid constituents, but usually contains about 12 parts of solid matter in 1,000 parts of the fluid in adults. This consists of a peculiar salivary matter called ptyalin, a fatty acid albumen, mucus, chlorides of potassium and sodium, soda, either free or in combination with albumen, lactates of potass and soda, phosphate and sulphate of soda, sulphocyanide of potassium, and the phosphates of lime, magnesia, and peroxyde of iron. This pure saliva is mixed in the mouth with the mucus secreted by its lining membrane, which renders the fluid more viscid. The researches of Dr. Wright show that saliva is capable of absorbing oxygen from the air in variable quantity, which sometimes exceeds, at others, falls short of half the bulk of the fluid ; but it also contains from one-eighth to one-twelfth of its volume of carbonic acid. The absorbed oxygen probably aids in the process of oxygenation. At least, Dr. Wright found that saliva remaining for some hours in a vessel filled with oxygen gas, was capable of converting a much larger quantity of starch into gum and sugar than an equal quantity of fresh saliva.

It is probable that healthy saliva judging from its composition and alkaline reaction, would exert very little if any action on the alloys employed in dentistry ; but the case is entirely altered when the saliva is in an unhealthy condition, and from being alkaline becomes acid. The alkaline character of healthy saliva is readily shown by placing a slip of reddened litmus paper on the tongue when the color is changed to blue ; but when this fluid becomes acid, blue litmus paper is reddened by contact with it. Donné states, that the saliva is acid in all cases of inflammation and irritation of the stomach ; in many cases of acute inflammation, such as pleurisy, inflammations of the brain, ague, rheumatic fever, and sometimes in mercurial salivation ; in fevers of a typhoid and inflammatory type, in measles previously to the eruption, and in miliary fever in which the saliva is said to be so acid as to cause a sensation of roughness of the teeth. To these might be added a long list of affections, accompanied by derangements of the digestive apparatus, in which acid saliva is secreted.

Donné states, that although he has never met with a single instance in which the saliva was acid, where the functions of the stomach were healthfully performed, nevertheless, some writers assert, and with truth, that acidity of the saliva does not invariably accompany derangement of the stomach, such as indigestion, especially when accompanied by, or dependent on, affections of the gastric nerves, in cases where the tongue is pale, flabby, tremulous and furred, with a bitter flavor in the mouth. In these cases the saliva is rarely acid, more frequently neutral, and often alkaline. The acidity of the saliva may depend on the presence of free lactic, acetic, muriatic, oxalic, or perhaps uric acid, but most frequently lactic acid. These acids, with the exception of the oxalic and uric, are present in healthy saliva, in combination with soda and potass; but when the bases are in insufficient quantity to saturate the acids, they of course will exist in a free state and give rise to acid reaction. Lactic acid appears to be in excess in the saliva of persons subject to gout, rheumatism, ague, diabetes, and in inflammatory conditions of the stomach and bowels; acetic acid in aphtha, scrofula, scurvy, small-pox, and protracted indigestion; also after the use of ascescent wines; accompanied by muriatic acid in simple gastric derangements from immoderate use of animal or vegetable indigestible food; but in these cases it is by no means improbable that the muriatic acid is derived from acid eructations from the stomach. Uric acid is said to have been found in the saliva of persons subject to gouty affections. Oxalic acid has been discovered in the saliva of those who are in the habit of eating large quantities of sorrel, (which contains oxalic acid,) or have taken large doses of liquor potassa. It is thought by some that free phosphoric acid will sometimes be discovered in it. Acidity of the saliva may be produced either by general or local causes. The acids may be secreted by the salivary fluids, either from direct depravation of function, or sympathetic with gastric disturbance; or again, acid may be excreted by these organs. Dr. Wright observes, that medicines and other substances taken internally, sometimes escape by the salivary glands, and in so doing impart to that secretion the properties they usually possess; that when minerals are absorbed into the system they usually escape from the body by the kidneys or skin, but sometimes they are discharged by the salivary apparatus, and mingling with the saliva, impart to it a stimulating and even an acrimonious quality; and he has known considerable local irritation to be produced by saline matter in the saliva, especially chloride of sodium, nitrate of potass, and iodide of potassium.

The saliva sometimes contains an excess of alkali which renders it acrid. Dr. Wright states, that in most of these cases, the natural alkali of the saliva is in excess; and that alcoholic liquids, aromatic, and other similar substances, are apt to escape from the system by the salivary glands, and in so doing, impart various degrees of acridity and offensiveness to the secretion. ' Morbid conditions of the saliva are not the only causes of decay of the teeth, and the corrosion of metallic alloys employed either for stopping them, or as plates for artificial teeth. Various articles of food retained between the teeth, or in the hollows, or in contact with the metallic plate or stoppings, such as vinegar and sugar, which by subsequent changes become acid, act with greater or less energy on the teeth. Again, the fluids which so commonly regurgitate from the stomach as acid eructations in dyspepsia and other gastric derangements, containing, as they do, a considerable portion of muriatic acid, corrode the teeth, as is evinced by the sensation of roughness so commonly felt in these cases.

From the preceding observations it is evident that, owing to the frequent changes of the saliva and gastric fluids, such substances should be selected for fixing artificial or stopping carious teeth, as cannot be acted on by the free acids or alkalies contained in these secretions. We must, therefore, direct our attention to the action of these agents on spurious alloys used for fixing artificial teeth, and the numerous amalgams, cements, enamels, &c., employed for stopping carious teeth. There is no department of the dental profession that has afforded so extensive a scope for quackery, imposition, and extortion, as that of supplying artificial and stopping carious teeth, while on no other department has so much ingenuity and mental labor been bestowed by the scientific dentist as on that of bringing these branches of his profession to perfection. Yet in none, perhaps, has the public, generally speaking, been more disappointed, in consequence of the extravagant and absurd pretensions of cheap advertisers, who profess to replenish the mouth with artificial masticators, and repair the dilapidation of carious teeth, with their poisonous compounds, as easily as a person could be furnished with a ready-made coat. Judging from the paragraphs in the newspapers and periodicals, it would seem that the public have been grossly deceived in trusting to those cheap advertisers as skillful and scientific practitioners, who use pure materials, innoxious to the health. Complaints of this nature are by no means new to respectable practitioners, most of whom could furnish scores of cases to substantiate their truth, while the records of police and county courts, when the dupes have had the moral courage to resist the imposition, will furnish ample testimony to the extortionate demands of these *soi-disant* cheap dentists. The letters and paragraphs which have appeared in the newspapers in reference to the inferior gold used by dentists, and its injurious effects, will, we doubt not, be productive of immense good to respectable practitioners; it will, however, be the means of directing public attention to a subject that seriously affects the general health, and personal appearance, of a considerable portion of the community; it will show how absurd are the pretensions, and how inferior must be the artistic skill, and how bad the materials of an artificial tooth fixed on gold, "*for five shillings*," which cannot be supplied by a conscientious or respectable practitioner, at a less cost than a guinea or upwards. There can be no question that either the person holding out the five shilling tariff, must use inferior materials and less skill, or the charges of the other must be an exorbitant imposition.

The time has now arrived for an explanation of these discrepancies in dentist's charges, since it would appear that the public has paid too dear a price for cheap dental operations at the expense of permanent local and constitutional disease, arising from the deleterious effects of bad adaptations and of those inferior metallic alloys, which suffer corrosion when in contact with the fluids of the mouth, and generate poisonous compounds.

It has been suggested, as a guarantee to the public of the purity of the gold used by dentists, that each article should be stamped at Goldsmith's Hall. To this proposition, a respectable practitioner can have no possible objection; but the application of the stamp is attended with insuperable difficulties.

To entitled the practitioner to the Company's mark, he must be licensed to sell gold, the punches must be registered at the Hall; and moreover, it is a rule with this City Company, never to stamp manufactured goods of any kind. The article must present the general form intended, with the springs, wire and rivets attached; the finishing, polishing, and fixing the teeth, may be done afterwards; but considerable force is necessary in stamping the work, by which such serious injury is done to the fitting of the plate, so that in all cases it would be necessary to re-model the frame work or basis.

The most simple, effective, and economical guarantee to the public would be, that every piece of work in gold should bear the *name* of the dental practitioner, and should any question arise regarding the purity of the material, the patient would only need to send it to the Assay Office, in Hatton Garden, for examination, with the results of which he would be furnished in two days, at a merely nominal cost, and without the least injury or deterioration of the artificial dental apparatus.

Having indicated a remedy for the evils that at present exist, we will proceed with our inquiries relative to the various metals used for forming the frame-work or basis of artificial, and the stopping carious teeth, with the substitution of one metal for another in cheap dentistry, and endeavor to ascertain to what extent the acids and alkalies of the saliva are likely to act, or enter into combination with, these metals or alloys, and form compounds which affect the health of the patient constitutionally and locally. The acids, as we have before stated above, existing in natural or diseased saliva, are the lactic, acetic, muriatic, and very rarely the oxalic and uric acids; the alkalies are potass and soda, chiefly the latter. The metals employed in the manufacture of the plates, wires, rivets and springs, upon which artificial teeth are mounted, are gold, platinum, palladium, silver and copper, either alone or alloyed in various proportions. Gold, from its incapability of being attacked by other agents than chlorine, undergoes no change or corrosive action from the constituents of the saliva; platinum, when pure, like gold, undergoes no change; palladium, a metal associated with platinum in its ores, likewise remains unacted on by the salivary fluids. It is not acted on by sulphuric or muriatic acids, but is oxydized and dissolved by nitric acid, and blackened by the hydro-sulphuret of ammonia. Pure silver resists the action of the acids and alkalines of the saliva, but its affinity for sulphur is so strong as to cause it to tarnish rapidly by contact with white of egg, or even exposure to the atmosphere, especially where coal fires are used. Pure copper is used only as an alloy with other metals for dental purposes, of which I shall treat more at large hereafter. It has a strong affinity for oxygen, and is gradually oxydized by exposure to to the air and salivary fluids, especially such as contain fatty matters; and is converted into a carbonate, which forms a greenish incrustation on the surface of the copper, and is readily dissolved by weak acids. Such is the chemical action of the acids contained in the saliva on the *pure* metals.

We now turn to the results of a similar action on the metallic alloys employed by dentists, in order to ascertain to what extent the acids of the saliva act upon them, and form deleterious salts. Alloys are, for the most part, more fusible than the pure metals of which they are composed. The metals, as a general rule, unite with each other in all proportions by fusion, but their mixture impairs the qualities of ductibility and malleability; but this, to a great extent, depends on the nature of the metals. The addition, for example, of one grain of lead to 2,000 grains of gold, diminishes its malleability to a surprising extent, but increases its hardness, so that in certain proportions the alloy of gold and lead is harder than that of gold and copper. The metals which are most destructive to malleability and ductility, are lead, antimony, arsenic and bismuth; a small proportion of copper deepens the color of gold and increases its hardness; a similar proportion of silver renders it more pale, but the alloy is harder than either of the pure metals. For the purpose of rendering gold harder and more capable of resisting wear and tear, a certain proportion of copper is legally added to the coinage. The tendency of many metals to combine with oxygen is increased when alloyed; but the reverse occurs with others. The action of acids on alloys, is modified by the properties and proportions of the respective metals. Gold alloyed with a very small proportion of silver, defends the latter from the action of nitric acid ; but when, as in the operation of quartation, the silver predominates, the whole of the latter is dissolved out by the acid, leaving the gold pure. Pure gold is never employed for the beds of artificial teeth, because it is so soft and flexible that it would very soon lose its shape, and thus become absolutely useless. The pure metal is only employed in the form of gold leaf for filling the cavities of carious teeth.

The standard gold for English coins contains about one-twelfth of its weight of copper, or copper and silver ; the hardness is increased by this admixture, without materially detracting from its malleability and ductility. The alloy with silver is tougher than that with copper. If the gold used by dentists was alloyed in the same proportion as that of our coinage, it would be too soft for its intended purposes, and would soon lose its shape ; still more so if alloyed with silver, independently of the color being paler than that associated with gold in the public mind. The gold employed by respectable practitioners for making plates, is never less than 18 carats pure, but more frequently nearly 19 ; which means that 24 parts of the alloy contain 18 parts of pure gold, with 6 of silver or copper. Platinum is not much used for plates, owing probably to the difficulty of procuring it pure, and to its color. Palladium is not often employed on account of its color, and the action of sulphur, which tinges its surface dark brown. Silver *never* ought to be employed, either alone or alloyed with copper, but only as we have already noticed for alloying gold. The standard silver for coinage consists of 13 parts of silver to 1 of copper ; but much more copper may be added without affecting its color. The copper is added to the standard for the same purpose as silver and copper to gold, that is, to increase its hardness, pure silver being too soft for this purpose. Silver becomes rapidly tarnished in the mouth, chiefly by the action of sulphur, which covers it with a brownish black crust. The sulphuret of silver thus produced possesses no dangerous or hurtful properties.

Copper, as we before observed, is only used for the purpose of alloying gold and silver, but if used in excess in either of the alloys, especially in the alloy with silver, it is powerfully acted on by the fluids of the mouth in the manner already described, forming absolutely poisonous compounds.

In order to test the relative effects of strong acids on the alloys of gold employed for plates for artificial teeth, the following experiments were made. The acid consisted of two parts of commercial nitric acid with one part of water. 24.47 grains of gold 14 carats fine, alloyed with silver, lost 1.15 grains by the action of the acid. 24.47 grains of gold 14 carats fine, alloyed with copper and silver, lost 8.42 grains. 24.97 grains of gold 14 carats fine, alloyed with copper alone, lost 4.77 grains.

These results are of great interest, because they show first, that gold alloyed with one metal, whether it be silver or copper, is less liable to corrosive action than an alloy which consists of the *three* metals, gold, silver and copper; secondly, that the alloy with silver alone resists the action better than that with copper alone.

The preceding observations demonstrate that no other metals or alloys than those of gold and platinum can be placed in the mouth without the liability to chemical changes, which either render the workmanship unsightly by changing its color, or yield noxious compounds from corrosion; and for these reasons no dental practitioner is justified in employing others than those named above.

Having determined the nature of the metals and alloys, our attention is next directed to still lower alloys, or combinations of metals in various proportions, used as solders, by which the rivets and springs are attached to the plates. These should approximate very closely to the standard alloy used as the basis for the plate itself; because when of a much lower standard, galvanic action is excited, and the more easily oxydizable metal is more rapidly acted on than in its pure state by the contact of the less easily oxydizable metal with the fluids of the mouth.

The alloy used as solder for fixing the rivets to the plate of 18 carats standard, should consist of 15 parts of fine gold, 4 of silver, and 4 of copper. This solder can only be used for gold of 18 carats, platinum or palladium, as any inferior alloy would be fused with the solder. This solder is not acted on by the acids or salts of the saliva. Silver solder is generally composed of silver and brass. This is more readily acted on by the fluids of the mouth than standard silver itself. The solder should in all cases assimilate in quality to the standard of the plate, for any great reduction would as a matter of course render the solder more liable to be acted on by the salivary fluids, and thus nothing would be gained in a sanitary point of view, by having only a part of the work of the proper standard, while the remainder had no higher value than eight or ten carats. The use of inferior solder affords ample means for chicanery and imposition in cheap mechanical dentistry and inferior artistic skill; the practice generally being to fabricate an extremely *thin* plate of the proper standard gold; the springs, wires, rivets, &c., averaging from 12 to 15 carats, and the solder employed for attaching them to the plate scarcely more than from 10 to 12 carats; or in round numbers, the whole piece of workmanship consisting of equal weights of pure gold and the baser metals. Were such a piece of dental manufacture submitted to the assay office, it would be discovered that the whole piece would not average more than from 12 to 14 carats, although the major part of the apparatus in appearance (the thin plate) bears the mark of respectability, and may probably approximate to the legal standard. It may be asked, why use an improper solder when the plate is of the proper standard? The answer is easy: the more gold is alloyed, the more easily does it fuse; and, therefore, if the highest quality of solder corresponding to the 18 carat plate is to be employed, the plate should be proportionately thick; and the springs, rivets, &c., of a higher standard, or the latter would fuse into a mass during the process of soldering, particularly where a large quantity of solder is used. The solder No. 1, used for the 18 carat standard plate, is rather more than 15 carats fine; No. 2 is about 13½ carats, and No. 3 rather more than 12 carats, fine.

Hitherto we have spoken of work bearing the appearance of respectability, although really very inferior. We shall now refer to the various substitutions and adulterations daily inserted into the mouths of patients, some of which are scarcely superior to pinchback or gilded copper, with the single exception, that for conscience sake they do contain a portion of gold. Pieces of work are frequently brought under our notice, in which the fittings of the teeth to the plate have not been properly made—a most important part of the work, absolutely essential to the comfort in wearing, and durability of the work—in which common pewter solder had been made to play the part of the mechanic in filling up the interstices, and fastening the springs and rivets. The substitution of one metal for another is very common in cheap dental work, and standard silver appears to be the favorite substitute. The plate is fashioned some way or some how; clasps, wires, rivets, &c., are attached with the *easiest* running solder—no matter what; either inferior silver or pewter, or both—the teeth fastened with sulphur, and then comes the climax and godsend to fraud and imposition—a coating of pure gold by the electrotype process, after which it is ready for insertion into the mouth. It is then forced some how or any how into its intended position, without any regard to carious or tender teeth, or the remains of diseased or irritating stumps; and in total ignorance the patient wears this spurious imitation—a compound of silver, sulphur, copper and verdigris—until local inflammation of the mucous membrane of the mouth, diseased gums, aphthous ulcers, accompanied by fetid breath, gastric and nervous derangement, discover to the wearer the old adage, that "all is not gold that glitters." Palladium, either alone or alloyed with silver, is acted on by the sulphur of the salivary fluids and food, and becomes blackened thereby; but the same remedy is applied by the cheap dentist, and a plate of this alloy is covered with a thin coating of gold by the aid of the galvanic battery, which, like charity, covers a multitude of dentists' sins.

Having explained above what *can be* and what *is* done in the way of sophistication and substitution of metals, I purpose making a few comments on the proper method of manufacturing a set of artificial teeth, and showing how far the actual cost of the raw material can coincide with the professed cheapness put forward, in which sets of teeth, "upon superfine gold," are offered at prices varying from £3 to £10, *finished* in the best style, and warranted to fit, or the money returned!!! Gold has been from time immemorial the admired and worshipped of man, from the golden image of Nebuchadnezzar down to the modern pieces bearing the portrait of our gracious queen; and the *auri sacra fames* has tempted statesmen, poets, nobles and philosophers to resort too frequently to mean and dishonorable arts for its acquisition. Gold holds its potent sway over the present generation as the key and stepping stone of ambition, and the food by which avarice and cupidity are nurtured. Chicanery and imposition are resorted to for its possession and its distribution, all being desirous of possessing this metal, either in their pockets or on their person, as jewelry, or in their mouths at the cheapest possible rate; and whether what they obtain be gold, or its imitations, the majority are satisfied with whatever wears its semblance, although when too late they sometimes discover that even *gold* may be bought too dearly.

The plates for a set or partial set of artificial teeth, should not only be of the proper standard, but of *sufficient substance and breadth to ensure strength and solidity* to the frame-work, when in use; and the solder employed in the fabrication, should, as I have before observed, assimilate in quality to the quality of the plate; the less solder used the better. These essentials in reference to the quantity and quality of the materials, involves a matter of importance in relation to the cost of the raw material—gold. It is well known, that the average price of standard gold is £3 15*s*. per oz., and any respectable practitioner will use for a full or a partial set, from an ounce to an ounce and three quarters, or even more of this metal, so that the gold itself, will, on the average, cost from £5 to £7; this it must be remembered, is independently of modeling, teeth, springs and fittings in the process of manufacture, to say nothing of artistic skill, finish and adjustment to the mouth, which plays an important part towards the ultimate success, ease, comfort, appearance and durability. Notwithstanding these facts, we have it daily announced by advertisements, handbills and placards, that whole sets of teeth can be furnished from £3 to £10, each person warranting his wares to be mounted on *pure, superfine and standard* gold, *with the highest mechanical finish and artistic skill*—his gold unalloyed, and himself the veritable Simon Pure. There can be only one answer to these impudent assertions, with reference to the quality and solidity of the materials employed. The cheap dental conjurer, if he does use pure materials, must have discovered the philosopher's stone, and be a transmuter of the baser metals, or he must have a California in his kitchen, or be a large shareholder in the Cornish copper mines, as well as an adept in electro-metallurgy. Much has been said and written in reference to the application of the Hall mark to the gold used by dentists; I have already shown that this mark would be of little value as a guarantee of the purity of the metals, unless, in an unmanufactured state; again, the silver mark may be substituted. The gold mark, or a private mark resembling that used for stamping German silver, *might* be employed, and the work covered with gold by the electro-type process, only those acquainted with the genuine London Hall marks would be able to detect the imposition, as the spurious might so closely resemble the genuine mark, as to deceive very good judges, unless compared with the latter. There are several other towns who have their halls or companies for stamping the precious metals, as Birmingham, Edinburgh, &c.

Jewelers and pawnbrokers frequently test the quality of gold without assaying it, by providing a number of points of gold, of different degrees of fineness, from the standard downwards, which are rubbed on a touchstone, and the streak compared with that produced by the article under trial. Nitric acid is also applied, and the quality of the gold judged of, by the amount of action on it by the acid. But I am of opinion, that the most certain test would be, as I have before advised, to send the suspected article to the Assay Office.

The metals employed for the plates, &c., for artificial teeth being disposed of, I shall conclude with some observations on the amalgams, and other compounds used for stopping decayed teeth.

There is quite as much, if not more, injury produced by the introduction of these substances into the mouth, as, with a few exceptions, they contain mercury in various proportions with other metals. Mercury with silver, forms a greyish white amalgam, soft, when first made, but becoming gradually hard in the mouth. This amalgam is readily acted on by the fluids of the mouth, and forms deleterious compounds, which act most injuriously on the alveolar membranes, gums and other parts of the mouth. Several cases are recorded, in which profuse salivation followed its use; and in other cases, where large cavities are thus filled, the patient becomes liable to pain, accompanied by a coppery taste in the mouth, especially, when any derangement of the stomach occurs. The chemical action on the amalgam is rendered more active by the galvanic circle produced by the contact of the two metals and the fluids of the mouth. This amalgam is advertised under the various names of mineral cement, mineral succedaneum, white enamel cement, new discovery, &c.: all are the same under different names. They are all composed of silver filings, or precipitated silver with zinc, made into paste with mercury.

Another compound employed for this purpose is fusible metal, composed of bismuth, lead and tin. It is applied by melting it over a candle or spirit lamp, and is inserted into the tooth before it solidifies. The temperature required to melt this alloy, is that of boiling water, and this would suffice to produce inflammation of the tooth; but the metal contracts on cooling, and no longer filling thc cavity of the tooth, admits the fluids of the mouth into the cavity, and exposes the whole surface of the metal to oxydation.

Lead, which is occasionally used, is readily acted on by the fluids of the mouth, and generates poisonous compounds. Silver leaf becomes quickly blackened, but the compounds of pure silver produced are innocuos. Melted sulphur is also used, but is liable to the same objections as fusible metal. Pure platinum might be used safely, but it is so much less ductile and malleable than gold, and its price so high that it can seldom, if ever, be employed with advantage. Pure tin, also, undergoes little change in the mouth; its compounds are innocuous, and may be employed with safety.

Vegetable cements are chiefly composed of solutions of gums, or gum resins, dissolved in spirit of wine. A compound of gutta percha has been introduced by Mr. Hill, an American dentist, which answers extremely well, and is wholly unacted on by the salivary fluids; but its color, a light brown, renders it objectionable for stopping front teeth. It is more available as a temporary stopping for sensitive teeth, until they are in a condition to bear stopping with gold.

Pure gold leaf is, however, the best and only material that ought to be employed for this purpose. Its toughness and durability, the ease with which it can be packed into the carious tooth, and its total resistance to the chemical action either of the saliva or food, mark it as especially fitted for stopping teeth.

We have thrown these remarks together, as much for the benefit and instruction of the public, and for their guidance in the choice of operators and materials, as for the information of our respectable professional brethren, at home and abroad; many of whom are unaware of the extent to which the substitution of inferior and deleterious materials is carried, to the detriment of the dental art in the eyes of the world.

To the public, who have been lately enlightened to some extent, by the publication in the newspapers of cases, in which serious injury, local and constitutional, has been inflicted on individuals, together with gross fraud upon their pockets, we commend the attentive perusal of the plain and impartial detail we have put before them, of the illegitimate practices resorted to by quacks and pretenders, and the proper and legitimate process, by which the skillful and intelligent dental practitioner, can hope successfully to supply the defects of age, accident, disease, or malformation. To those who have been already sufferers from the arts and tricks of the unscrupulous and dishonest—to those who have already been in the hands of the Philistines, we need scarcely impress the fact, that the best advice and the best materials, the best workmanship and the guarantee of a respectable practitioner, will, in the long run, be found the true economy. Upon those who are yet deliberating as to their first choice of dental assistance, we would strongly urge the advisibility of eschewing all who announce to the world miracles of art, or miracles of cheapness, and who hold forth the accomplishment of the impossible and impracticable, as baits to entrap the ignorant and unwary. We feel satisfied, that in the foregoing statements we have advanced nothing but what is true, and nothing that can give offence to the skillful and educated practitioner; while to those who have no claims to either, we need offer no apology for the exposure of unfounded pretensions and professional deception. We have no sinister or selfish objects in view, and if our statements shall have had the effect of enlightening the public mind upon a very important department of surgical and mechanical science, we shall feel ourselves amply rewarded.

